# Genomic analyses revealed low genetic variation in the intron-exon boundary of the *doublesex* gene within the natural populations of *An. gambiae* s.l. in Burkina Faso

**DOI:** 10.1186/s12864-024-11127-y

**Published:** 2024-12-18

**Authors:** Mahamadi Kientega, Ioanna Morianou, Nouhoun Traoré, Nace Kranjc, Honorine Kaboré, Odette N Zongo, Abdoul-Azize Millogo, Patric Stephane Epopa, Franck A. Yao, Adrien M G Belem, Austin Burt, Abdoulaye Diabaté

**Affiliations:** 1 Institut de Recherche en Sciences de la Santé, Bobo-Dioulasso, 01 BP 545 Burkina Faso; 2 Department of Life Sciences, Imperial College London, London, SW7 2AZ UK; 3 Université Nazi BONI, Bobo-Dioulasso, 01 BP 1091 Burkina Faso; 4 University of East Anglia, Norwich Research Park, Norwich, NR4 7UH UK; 5 Institut des Sciences des Sociétés, Ouagadougou, 03 BP 7047 Burkina Faso

**Keywords:** Gene drive, *Doublesex gene*, Genetic polymorphism, *An. Gambiae* s.l

## Abstract

**Background:**

The recent success of a population control gene drive targeting the *doublesex* gene in *Anopheles gambiae* paved the way for developing self-sustaining and self-limiting genetic control strategies targeting the sex determination pathway to reduce and/or distort the reproductive capacity of insect vectors. However, targeting these genes for genetic control requires a better understanding of their genetic variation in natural populations to ensure effective gene drive spread. Using whole genome sequencing (WGS) data from the Ag1000G project (Ag3.0, 3.4 and 3.8), and Illumina pooled amplicon sequencing, we investigated the genetic polymorphism of the intron-4–exon-5 boundary of the *doublesex* gene in the natural populations of *An. gambiae sensu lato* (s.l.).

**Results:**

The analyses showed a very low variant density at the gRNA target sequence of the Ag(QFS)1 gene drive (previously called *dsxF*^*CRISPRh*^) within the populations of West and East Africa. However, populations from the forest area in Central Africa exhibited four SNP at frequencies ranging from 0.011 to 0.26. The SNP (2R:48714641[C > T]) at high frequencies, i.e. 0.26 is identified within the *An. coluzzii* population from Angola. The analyses also identified 90 low frequency (1 − 5%) SNPs in the genomic region around the gRNA target sequence (intron-4–exon-5 boundary). Three of these SNPs (2R:48714472 A > T; 2R:48714486 C > A; 2R:48714516 C > T) were observed at frequencies higher than 5% in the UTR region of the *doublesex* gene. The results also showed a very low variant density and constant nucleotide diversity over a five-year survey in natural *An. gambiae* s.l. populations of Burkina Faso.

**Conclusion:**

These findings will guide the implementation of *doublesex*-targeted gene drives to support the current control tools in malaria elimination efforts. Our methods can be applied to efficiently monitor the evolution of any sequence of interest in a natural population via pooled amplicon sequencing, surpassing the need for WGS.

**Supplementary Information:**

The online version contains supplementary material available at 10.1186/s12864-024-11127-y.

## Background

Malaria is the deadliest vector-borne disease in the world, caused by *Plasmodium* parasites transmitted to humans by female *Anopheles* mosquitoes. According to the WHO, deaths attributed to malaria have increased from ~ 400,000 (2018) to ~ 600,000 (2022) in the past five years [1]. This has been primarily attributed to the spread of insecticide resistance across sub-Saharan Africa, where the disease is endemic. In response to these challenges, innovative strategies based on genetic control are being developed to strengthen current tools and accelerate malaria elimination [2]. Homing-based gene drives are engineered selfish genetic elements propagating their inheritance using a homing endonuclease [3]. Gene drives can be used for vector control by being engineered to spread desirable genetic traits in the population, such as parasite refractoriness (population replacement), or by spreading negative fitness traits, such as infertility, amongst vector populations (population suppression). The CRISPR/Cas9 gene editing system has been adapted to act as an RNA-guided homing endonuclease for use in gene drive strategies by inserting the Cas9 and gRNA cassettes within their recognition sequence [4] (Supplementary Material 3). CRISPR-based gene drives have shown great promise as tools to combat malaria, both as population replacement [5] and population suppression genetic control strategies [6]. Gene drive strategies differ from traditional genetic modification methods because of their ability to rapidly self-propagate, their inheritance bias, and their potential for population-level change.

Most population replacement strategies are focused on introducing genetic modifications or novel transgenes to make the new population pathogen-refractory [7, 8]. Previous studies have shown that FREP1 mediates *Plasmodium* invasion of the *Anopheles* midgut epithelium [9]. CRISPR knock-outs of the *FREP1* gene showed resistance to human and rodent malaria parasites [10]. Similarly, the expression of small antimicrobial peptides in the mosquito midgut delayed the sporogonic development of the malaria parasite. It could be propagated in a single-generation experiment through gene drive homing [5].

Conversely, population suppression strategies aim to alter key population growth parameters, such as sex ratio and fitness. The sex chromosomes of *An. gambiae* were targeted by a synthetic sex-distorter I-PpoI, which is not a gene drive, and designed to induce a strong negative bias toward X chromosome–carrying spermatozoa, resulting in 95% male offspring [11]. Similar rates were also achieved using a synthetic CRISPR/Cas9-based sex distorter [12]. Recent studies have shown additional benefits in targeting the sex determination pathway to disrupt the sex ratio in the offspring for vector control purposes [13, 14].

In most insect species, the sex determination pathway starts with a primary central gene that stimulates the molecular cascade, leading to the alternative splicing of the *Doublesex* (*dsx*) and *Fruitless* (*fru*) genes [15], making these two genes the endpoint of the sex determination mechanisms [16]. The *Doublesex* gene (*dsx*) plays a critical role in the sexual differentiation of a wide range of insects, particularly the fruit fly *Drosophila melanogaster*, where its role in sex determination has been extensively studied. The architecture and function of the *dsx* gene have been well characterized in various insects [17, 18], including *Anopheles* mosquitoes [19]. In *An. gambiae*, the *dsx* gene spans an 88.598 kb (2R:48703664–48792262) sequence on chromosome 2R and consists of seven exons, with exon five being female-specific and exon six male-specific. Alternative splicing of the *dsx* gene during embryonic development produces two isoforms based on the sex-specific exons, which control the expression of endpoint genes required to exhibit physical sexual dimorphism [20].

Using CRISPR/Cas9, the disruption of the female-specific intron-4-exon-5 boundary of the *dsx* gene in *An. gambiae* resulted in morphological abnormalities in homozygous knock-out females, including in the development of the proboscis, which caused knockout females to be unable to draw a blood meal, mate, and produce offspring [6]. A CRISPR-based gene drive built against the female isoform of *dsx* (Supplementary Material 3B), termed Ag(QFS)1 (previously called *dsxF*^*CRISPRh*^), was able to rapidly spread in caged laboratory populations, causing a swift reduction of vector population density, and ultimately eliminating the populations within a year [6, 14, 21]. This success made the *dsx* gene a relevant target for developing genetic control strategies to reduce the population density of malaria mosquitoes and other harmful insects, including agricultural pests [22].

Although gene drive targeting the *dsx* gene is a promising tool for vector control, its success in wild populations requires that the target site be very little polymorphic. The presence of genetic polymorphisms in a gene drive target sequence could inhibit gRNA recognition and subsequent Cas9 cleavage, limiting the spread of the homing and consequently reducing the efficacy of the genetic tool [23–25]. Therefore, the success of most CRISPR/Cas9-based genetic control tools requires the target sequence to show limited genetic variation in natural populations. This study investigates the genetic variation within the intron-4-exon-5 boundary (2R: 48714420–48714720) of the *Doublesex* gene within wild *An. gambiae* s.l. by analyzing existing population genomics data generated by the *Anopheles gambiae* 1000 genomes project (Ag1000G), as well as newly generated data through targeted pooled amplicon sequencing of wild-caught mosquito populations from Burkina Faso. We also investigate the evolution and spatial distribution of the genetic polymorphisms discovered. We reveal the limited variation of the female-specific intron-exon boundary of the *doublesex* gene in natural populations. These results are valuable in guiding the implementation of gene drive tools to complement malaria elimination efforts in Africa.

## Results

### Spatial distribution of genetic variants in the Ag(QFS)1 gene drive target sequence

**Fig. 1:**
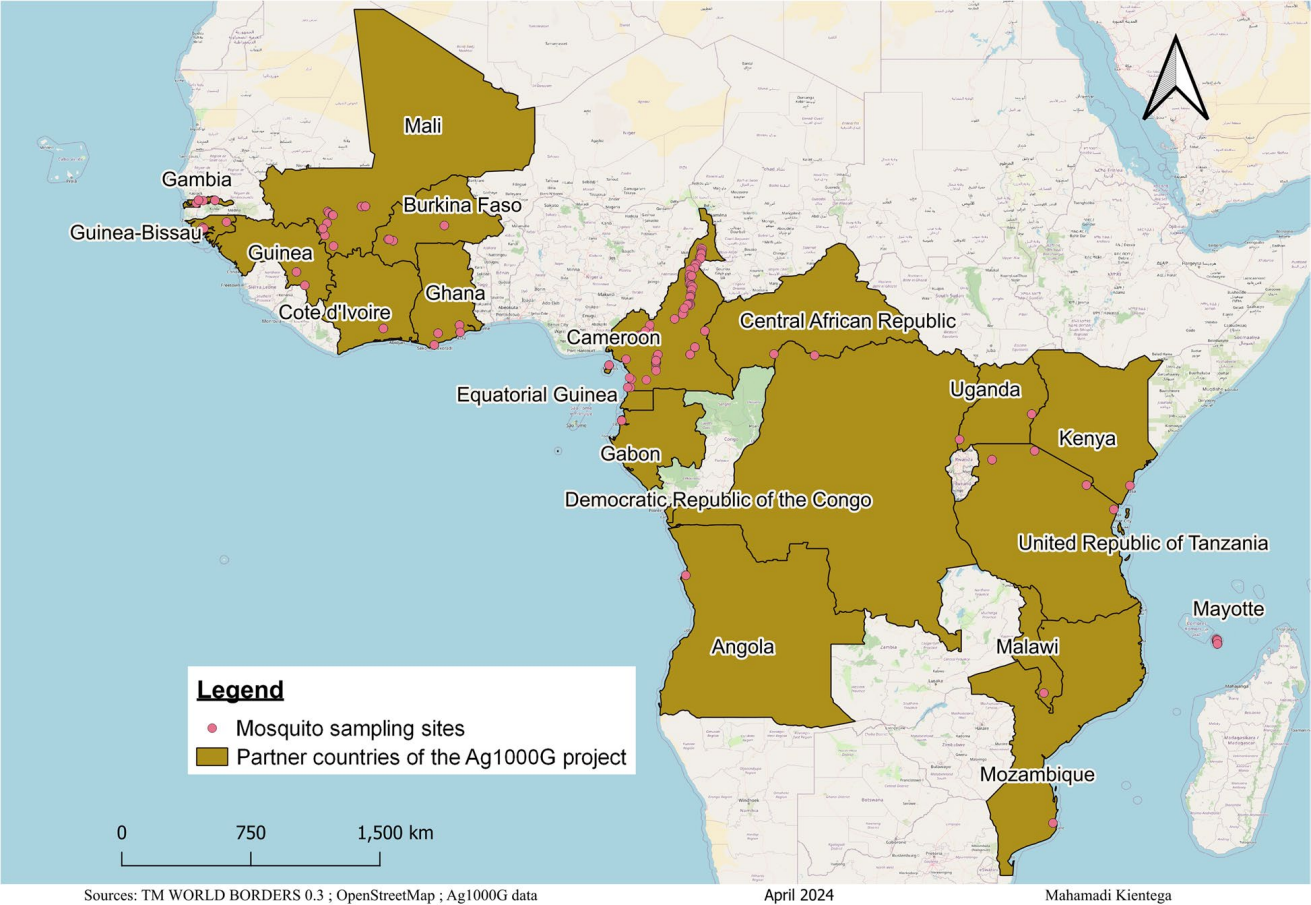
Location of sampling sites in the 19 African countries where *Anopheles* mosquitoes were collected for the *Anopheles gambiae* 1000 Genomes Project

We first investigated the distribution of variants across a sequence spanning the intron-4–exon-5 boundary of the *doublesex* gene (2R: 48714420–48714720), including the Ag(QFS)1 gene drive target sequence (2R: 48714637–48714660) using an existing genomics dataset generated by the *Anopheles gambiae* 1000 genomes Consortium (Ag1000G). This dataset includes SNP calls from 4200 wild-caught *An. gambiae* s.l. (*An. coluzzii*,* An. gambiae* s.s. and *An. arabiensis*) mosquitoes from 19 African countries (Fig. 1).

We identified 143 single nucleotide polymorphisms (SNPs) at varying frequencies from 0.000609 to 0.59 in the intron-4–exon-5 boundary of the *doublesex* gene within vector populations collected in 17 African countries (Supplementary Material 1). The SNP 2R:48,714,486[C > A], located in the UTR sequence of the exon 5, is found at relatively high frequencies (freq. ~ 0.12–0.59) in all *An. coluzzii* populations. The third allele of 2R:48,714,486 ([C > G]) is only identified in *An. arabiensis* populations (Freq. ~ 0.006–0.15).

**Fig. 2:**
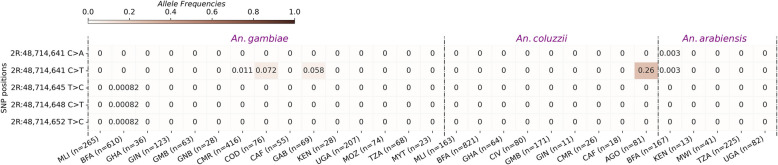
Heat map showing the allele frequencies of the variants in the Ag(QFS)1 gene drive target sequence (2R: 48714637–48714660) within the *An. gambiae* s.l. populations collected in 19 African countries; the y-axis of the heat map shows the non-synonymous variant positions in the 2R chromosome, while the x-axis shows the populations of *An. gambiae* s.l. and number of individual mosquitoes processed (n); the gradient color bar shows the distribution of the allele frequencies; MLI: Mali; BFA: Burkina Faso; AGO: Angola; CAF: Central African Republic; CIV: Côte d’Ivoire; CMR: Cameroon; COD: Democratic Republic of Congo; MYA: Mayotte Island; GAB: Gabon; GHA: Ghana; GIN: Guinea; GMB: Gambia; GNB: Guinea-Bissau; KEN: Kenya, MOZ: Mozambique; MWI: Malawi; TZA: Tanzania; UGA: Uganda

The target sequence of the Ag(QFS)1 gene drive is located in the boundary of the intron 4 and the exon 5, spanning 23 bp from the nucleotide in the 2R chromosome. This sequence contained five SNPs in the vector populations at relatively low frequencies (freq. ~ 0.00–0.26). Most SNPs were identified at very low frequencies (Freq. < 0.01) in West Africa and the *An. gambiae* populations of Burkina Faso, while no SNP was identified in the populations of East Africa (Fig. 2). The most abundant target site SNP, 2R: 48,714,641[C > T], was found at frequencies higher than 0.05 (0.011–0.26) in the *An. gambiae* populations of central Africa. Specifically, the 2R: 48,714,641[C > T] SNP was found at frequencies of 0.26 in Angola, 0.07 in the DRC, 0.06 in Cameroon, and 0.01 in Gabon. This was previously reported as a G > A SNP, as read on the antisense DNA strand [6, 26]. This position was found to be triallelic (1 reference and 2 alternative alleles), and its third allele, 2R: 48,714,641[C > A], was found in *An. arabiensis* populations from Burkina Faso at a frequency lower than 0.01 (Fig. 2). The presence of this SNP (2R: 48714641[C > T]) at frequencies higher than 5% in the gRNA target sequence within the Central African populations raised concern about the spread of the Ag(QFS)1 gene drive in these populations. However, it was found to be cleavable by the Cas9/gRNA ribonucleoprotein in vitro [6], and by the Ag(QFS)1 gene drive in vivo [26], so the gene drive should be able to spread in its presence. No SNPs were identified in the other *An. gambiae* populations of West and East Africa.

## Time series variation of the gene drive target sequence in Burkina Faso

Using the Ag1000G dataset, we also investigated the year-to-year genetic variation in the intron-4–exon-5 boundary of the *doublesex* gene within wild *An. gambiae* s.l. populations collected from 2012 to 2019 in three villages (Bana, Souroukoudinga and Pala) of Burkina Faso. The analyses identified 90 SNPs at frequencies ranging from 0.001 to 0.54 in the intron-4–exon-5 boundary of the *doublesex* gene over the seven-year survey (mainly in the untranslated region, UTR of *doublesex*) (Supplementary Material 2). The 2R:48,714,486[C > A] SNP, present in the *dsx* exon 5 UTR, was the most abundant SNP identified at frequencies of 0.39–0.54. All other SNPs were found at frequencies lower than 0.01. Most of the SNPs (except 2R:48714445[C > A,T], 2R:48714453[G > A], 2R:48714692[T > A] in *An. gambiae* s.s. and 2R:48714486[C > A] in *An. coluzzii*) were non-constant over the years, i.e. they appeared and disappeared the following year, presumably removed by purifying selection or drift.

**Fig. 3:**
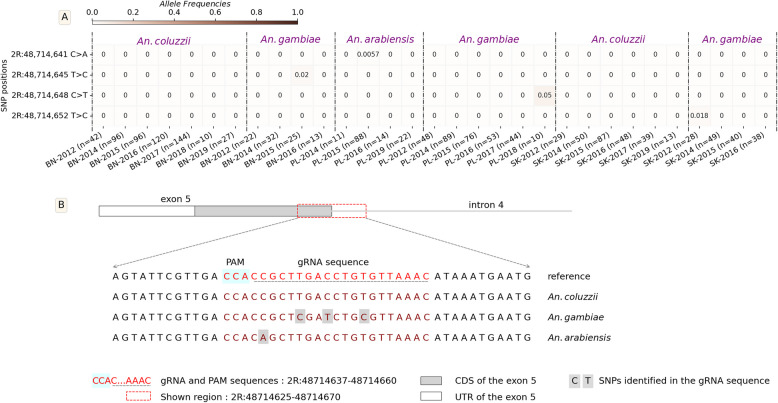
Allele frequencies of the SNPs in the gene drive target sequence (2R: 48714637–48714660) within the *An. gambiae* s.l. populations collected in 3 villages (Bana, Pala, and Souroukoudinga) from 2012 to 2019 in Burkina Faso; A: Heat map showing the evolution of the allelic frequencies of the SNPs in the gRNA target sequence; the y-axis of the heat map shows the positions of non-synonymous variants in the 2R chromosome while the x-axis shows the populations of *An. gambiae* s.l. and several individual mosquitoes processed (n). The gradient color bar shows the distribution of the allele frequencies. BN: Bana; SK: Souroukoudinga; PL: Pala. B: mapping of the SNPs positions alongside the reference genome in the different populations; PAM: protospacer adjacent motif

The Ag(QFS)1 gene drive target sequence (2R:48714637–2R:48714660) displayed four non-constant SNPs at very low frequencies (~ 0.0057–0.05) from 2012 to 2019 in the vector populations. These SNPs were non-constant over the seven-year survey and each of them was removed in the following year after its apparition in the populations (Fig. 3).

**Fig. 4:**
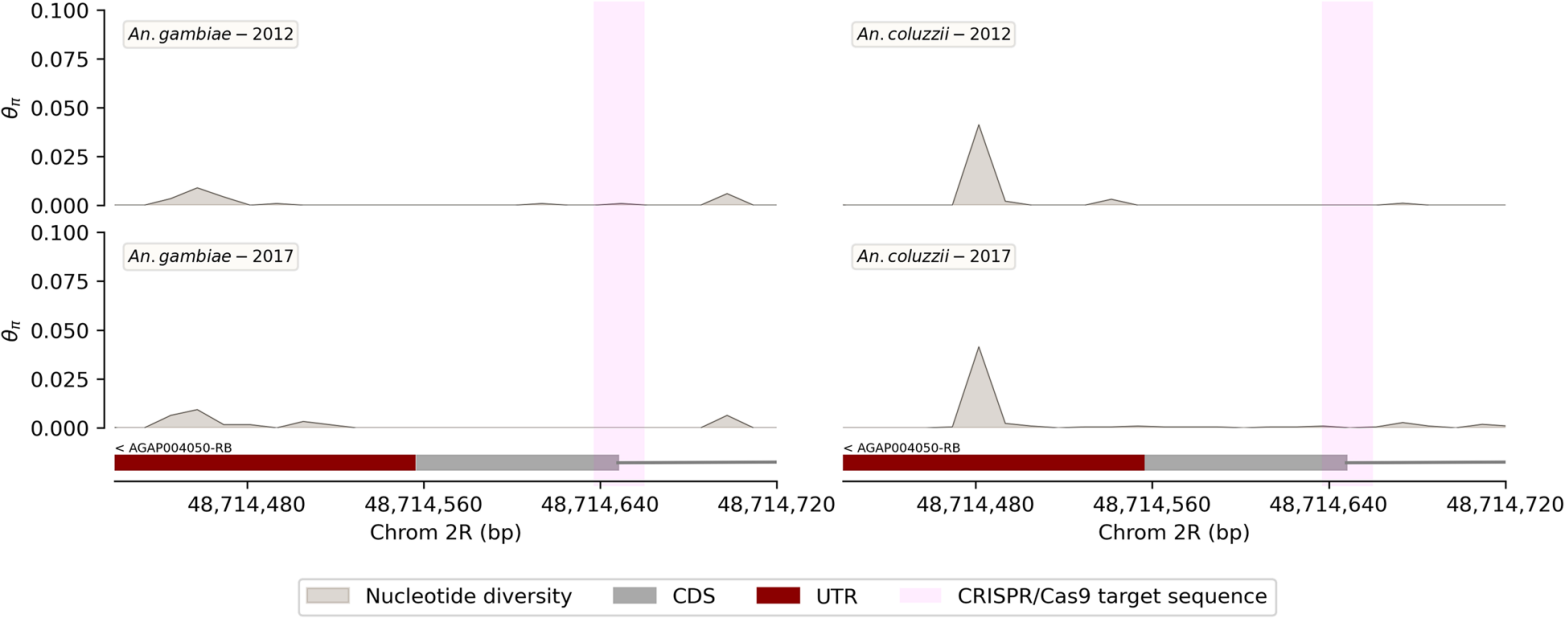
Evolution of the nucleotide diversity in the *An. gambiae* s.s. and *An. coluzzii* populations collected in 3 villages (Bana, Pala and Souroukoudinga) from 2012 to 2017 in Burkina Faso; the figure on top represents samples collected in 2012 while the figure below represents samples collected in 2017; the rectangles on the bottom shows the intron-4-exon-5 boundary of the *doublesex* gene (dark red: exon-5 untranslated sequence; gray: exon-5 translated sequence and simple gray line: intron-4); the two vertical pink-filled spans are delimiting the Ag(QFS)1 or CRISPR/Cas9 target sequence; the y-axis of the figures shows the nucleotide diversity (*θ*_*π*_) while the x-axis shows the 2R chromosome positions; the nucleotide diversity was constant over the five years and the high values were recorded in the UTR region but remained steady over the five years

The nucleotide diversity (*θ*_*π*_) ranged from 0 to 0.04 in the female-specific intron-exon boundary of the *doublesex* gene. Interestingly, the nucleotide diversity was constant over the years (from 2012 to 2017). The highest nucleotide diversity (*θ*_*π*_ *~ 0.04*) was recorded in the UTR region of the female-specific intron-exon boundary of the *doublesex* gene. In the target sequence of the Ag(QFS)1 gene drive, the nucleotide diversity was close to 0 in 2012. It remained so until 2017 (Fig. 4). The analyses showed a constant nucleotide diversity in the gRNA target sequence in the *An. gambiae* population of the sampling area. One of the challenges of genetic control remains the potential emergence of new genetic variants at the target sequence, over the time, through evolutionary processes. Our results showed no change in the genetic variation of the gRNA target sequence within the natural populations of the three villages (Bana, Pala and Souroukoudinga) over the seven-year survey, which is promising for the long term efficacy and spread of a gene drive strategy targeting this sequence.

## Pooled amplicon sequencing to detect genetic variants in the Ag(QFS)1 gene drive target sequence

The application of cost-effective and efficient methods is essential for monitoring the evolution of the Ag(QFS)1 gene drive target sequence in the natural population. Here, we employed pooled amplicon sequencing to investigate the genetic polymorphism of the Ag(QFS)1 gene drive target sequence in natural populations from Burkina Faso.

**Fig. 5:**
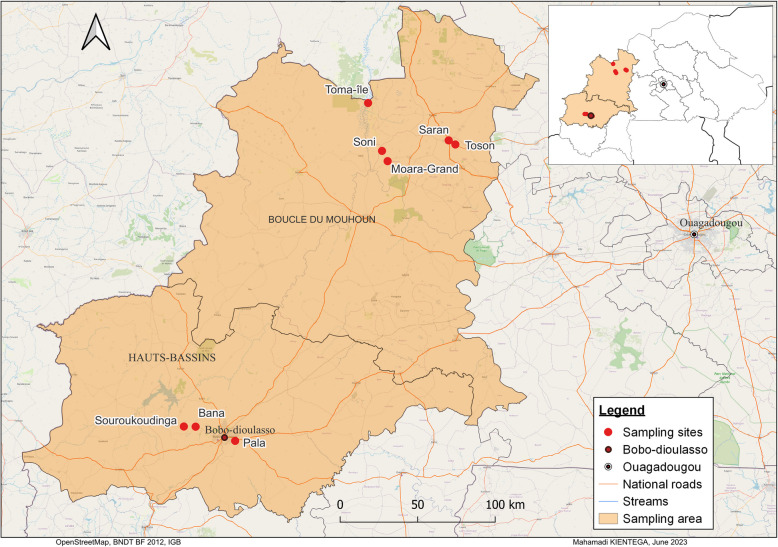
Mosquito sampling sites

We genotyped more than 600 individual mosquitoes belonging to three species of the *An. gambiae species* complex (*An. gambiae s.s.*,* An. coluzzii* and *An. arabiensis*) sampled in Bana, Pala, Souroukoudinga, Soni, Moara-Grand, Toma-Ile, Toson, Saran and from laboratory colony (Fig. 5). *An. coluzzii* was the most predominant species in almost all the villages except Pala, where *An. gambiae* s.s. and *An. arabiensis* were most prevalent. This allowed the formation of 12 pools (9 pools of *An. coluzzii*, 2 pools of *An. gambiae* s.s. and 1 pool of *An. arabiensis*) of 50 individuals for gDNA extraction and pooled amplicon sequencing of a 365 bp region encompassing the intron4-exon5 boundary of *doublesex*, as well as the whole exon 5 CDS, including the Ag(QFS)1 target site. The genomic data analyses identified 60 variants, compared to 67 variants detected in the Ag1000G data in the same 365 bp region. Of the 60 SNPs, 17 positions were found to be triallelic. Importantly, no indels were identified in the dataset. Most of the variants (*n* = 49 SNPs) were identified in the *An. coluzzii* populations followed by *An. gambiae* s.s. (*n* = 34 SNPs) and *An. arabiensis* (*n* = 16 SNPs), which showed this region’s lowest degree of genetic diversity. The SNP density was approximately 0.2 bp^−1^ [60/300], indicating the presence of 1 SNP per 5 bp. Most of these SNPs were identified in the UTR of exon 5 and intron 4, and they are expected to show higher variation as they constitute non-coding regions. The exon 5 CDS showed a varying number of SNPs between the populations, from 1 SNP (0.011 bp^−1^ [1/91]) in the laboratory colony to 7 SNPs (0.08 bp^−1^ [7/91]) in the *An. gambiae* s.s. samples of Pala. Only two of the SNPs (2R:48714617(C > T) and 2R:48714592(C > T)) identified in the exon 5 CDS were constant and present in all wild-caught populations.

**Fig. 6:**
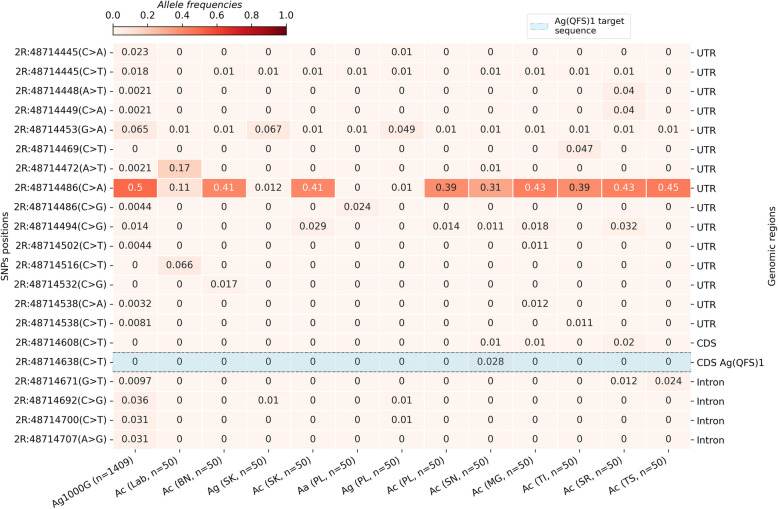
Heat map showing the allele frequencies of the SNPs (whose max frequencies are higher than 1%) identified in the female-specific intron-exon boundary of the *doublesex* gene identified via pooled amplicon sequencing from laboratory (Lab) and wild-caught mosquito samples (BN: Bana village, SK: Souroukoudinga, PL: Pala, SN: Soni, MG: Moara-Grand, TI: Toma-île, SR: Saran, TS: Toson, Ac: *An. coluzzii*, Ag: *An. gambiae* s.s., Aa: *An. arabiensis*); SNPs were called using the lofreq software; the y-axis of the heat map shows the positions of the non-synonymous variant in the 2R chromosome while the x-axis shows the populations of *An. gambiae* s.l. and number of individual mosquitoes processed (n); the gradient color bar shows the distribution of the allele frequencies; the sky blue band shows the frequencies of the SNPs identified in the Ag(QFS)1 or CRISPR/Cas9 target sequence; UTR: the untranslated region (2R:48714420–48714556) of the female-specific exon5; CDS: the coding sequence (2R:48714557–48714648) of the female-specific exon5; Intron: a part (2R:48714649–48714720) of the female-specific intron4; Ag1000G: *Anopheles gambiae* 1000 genomes

**Fig. 7:**
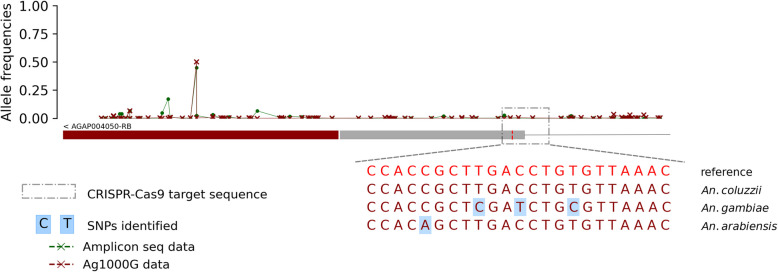
Distribution of the SNPs and their frequencies alongside the intron-4–exon-5 boundary of the *doublesex* gene; the rectangle on bottom shows the intron-4-exon-5 boundary of the *doublesex* gene (dark red: exon-5 untranslated sequence, gray: exon-5 translated sequence and simple gray line: intron-4); the y-axis shows the allele frequencies of the SNPs and the x-axis shows the positions of the SNPs in the 2R chromosome; the two vertical dashed lines are delimiting the Ag(QFS)1 or CRISPR/Cas9 target sequence; The DNA sequences at the bottom shows the SNPs positions alongside the reference genome in the gRNA target sequence within different populations. GD: « Gene Drive »; Amplicon seq. data: Amplicon sequencing data from laboratory (Lab) and wild-caught mosquito samples from 8 villages in Burkina Faso; Ag1000G data: Whole genome sequencing data of *Anopheles gambiae* 1000 genomes Consortium from wild caught *An. gambiae* s.l. mosquitoes collected in 3 villages from Burkina Faso

Almost all SNPs were identified at low frequencies (Freq < 0.05) except the SNP 2R:48,714,486(C > A) identified in the UTR sequence of the exon 5 at relatively high frequencies (freq = 0.31–0.45) in the *An. coluzzii* populations, while in *An. arabiensis* and *An. gambiae* s.s. it was present at low frequencies (freq. ~ 0.01). A third allele of this position, the SNP 2R:48,714,486(C > G), was identified at low frequencies (freq. = 0.024) in *An. arabiensis* populations. In the laboratory samples, this SNP is found at low frequencies and seems to be replaced by the SNP 2R:48,714,472 (A > T), also identified in the UTR sequence of exon 5 at a frequency of 0.17. Fig. 6 shows the allele frequencies of the SNPs whose maximum frequencies were higher than 0.01 in at least one population. The Ag(QFS)1 gene drive target sequence exhibited one SNP at low frequencies, i.e. less than 0.05. The analyses showed most of the SNPs in the UTR sequence of the exon 5 of the *dsx* gene, especially those with relatively high frequencies (Fig. 7). Interestingly, the distribution of the SNPs along the target sequence of the *dsx* gene and their allele frequencies were similar to those found in the time series Ag1000G data in Burkina Faso.

## Discussion

Malaria control remains a major challenge in sub-Saharan Africa due to the rapid spread of resistance. In response to this challenge, innovative control strategies are being developed to strengthen current tools and accelerate the elimination of malaria [2]. Ongoing genetic engineering and biocontrol studies through endosymbionts intend to develop effective and sustainable control tools to reduce malaria transmission [27, 28]. By targeting the sexual determination pathway [6], CRISPR-based gene drive technologies demonstrated their potential advantages as the basis for developing effective and sustainable tools to support malaria elimination efforts [7]. However, the success of a CRISPR-based gene drive for vector control requires its target sequence to show as little polymorphism as possible in natural populations to ensure efficient homing rates. Another possible prerequisite is for the target sequence to be highly conserved over the years to ensure the continued matching of the gRNA to the target sequence in natural populations several years after the release [29]. This study investigated the genetic polymorphism of the intron-4-exon-5 boundary of the *doublesex* gene within the natural populations of *An. gambiae* s.l. These insights may contribute for the future deployment of gene drive control strategies targeting the *doublesex* gene [6, 14].

The results showed a variable genetic polymorphism among the vector populations with a global variant density of one SNP every 5 bp. This density was low compared to what was previously found in the *dsx* gene and in the *An. gambiae* genome that was about one SNP every two nucleotides [30, 31]. Interestingly, most variants were identified in the gene’s untranslated region of exon 5. The analysis displayed a meager polymorphism in the Ag(QFS)1 target sequence (2R: 48714637–48714660). Analyses of time series data in Burkina showed four non-constant SNPs over a seven-year survey in the Ag(QFS)1 target sequence. Each SNPs appeared in different years and were removed the following year after their occurrence in the same population, presumably removed by purifying selection or drift. This pattern of genetic variation in the target sequence is expected to be sufficient for the rapid spread of the Ag(QFS)1 gene drive in natural populations. However, given that natural populations are dynamic, the genetic variation might be different in space over time. This situation is observed in the forest area of Central Africa where *An. gambiae* s.l. populations exhibited an SNP 2R:48,714,641[C > T] at frequencies reaching 0.26 in the gRNA target sequence in Angola, Democratic Republic of Congo, Cameroon, and Gabon but not in the Central African Republic. The third allele of this position, the 2R:48,714,641[C > A] was found in *An. arabianesis* populations of Burkina Faso. The SNP 2R:48,714,641[C > T] was identified in previous studies at Hardy-Weinberg equilibrium in *An. coluzzii* and *An. gambiae* populations of Angola [6, 26]. The emergence pattern of this SNP suggests its ubiquitous presence at evolving frequencies in the forest area of Central Africa and its presence could reduce the spread of the *dsx-drive* in vector populations. Recent studies showed a complex genetic structure of *An. gambiae* populations in Africa with important variants flow between populations from Western, Central and Eastern Africa [30, 31]. The *kdr* mutations (*kdr-L995F* and *kdr-L995S*) constitute an example of continent-widespread genetic variants in the *An. gambiae* population [32]. Seeing the high connectivity of *An. gambiae* populations [30, 31], the spread of the Cas9-based *dsx-drive* could be reduced if the SNP 2R:48,714,641[C > A] is positively selected and increased in frequency in the populations. Thus, the evolutionary processes driving this variant need to be investigated to understand the extent and the evolution of this SNP within the vector populations in Central Africa.

The emergence of resistance alleles remains a significant challenge in developing Cas9-based vector control tools [25, 33]. In addition to the existing variants, new alleles resulting from de novo mutations could be selected, threatening the efficacy of CRISPR-based gene drives in natural populations. Resistance variants may be introduced by gene drive activity upon error-prone NHEJ repair of a Cas9-induced double-stranded break in the DNA. This can be exacerbated if the gene drive allows Cas9 deposition into the early embryo, where NHEJ occurs preferentially over HDR [25, 34, 35]. Several strategies are being explored to reduce the potential emergence of resistance variants in natural populations. Using alternative promoters to contain Cas9 spatiotemporal expression in germline tissues reduced the rate of resistant allele creation [25].

Moreover, using multiplexed gRNAs targeting neighboring sequences simultaneously constitutes a promising strategy [26, 36, 37]. In this case, if a resistant allele gets created or is pre-existing in the population at one of the target sites, it will be removed as long as at least one other target site is still cleavable, and able to induce homing [26]. Resistant alleles would need to co-exist at all target sites to prevent gene drive homing, and they will need to cooperatively encode a functional gene product to prevent long-term gene drive spread. Nevertheless, this strategy remains complex in practice because of the difficulties in finding many gRNAs targeting neighboring sites and exhibiting suitable parameters in a single gene [38]. Another approach to prevent the emergence of resistance could be using engineered endonucleases or alternative nucleases such as dCas9-FokI and Cpf1/Cas12a. These nucleases showed an ability to tolerate target sequence variation, so existing polymorphisms at the cut site or outside this site are less likely to prevent cleavage and homing [39, 40]. Recently, a Cas12a split gene drive was developed in *Drosophila melanogaster* [41]. Combining a homing gene drive with an autosomal sex-distorter would also reduce the likelihood of a homing-resistant allele being selected [14]. Ultimately, all alternative strategies developed to mitigate resistance would only be successful if the gene drive targets a highly conserved sequence with minimal polymorphism tolerance. The present study set out a framework for in-depth investigation of the pre-existing natural variation in a natural population to inform gene drive implementation. Future directions would continue to monitor the evolution of genetic variation in the gene drive target site within natural vector populations and search for the potential presence of gene drive target sites in the genome of secondary vectors.

## Conclusion

In this study, we investigated the genetic polymorphism of the intron-4-exon-5 boundary of the *doublesex* gene, a potential target for developing Cas9-based control tools. The results showed very low polymorphism at the target sequence of the Ag(QFS)1 gene drive in the anopheline populations of Burkina Faso. No SNP was found under positive selection in this region over a seven-year (from 2012 to 2019) survey in *An. gambiae* s.l. populations from Burkina. Interestingly, most vector populations in West and East Africa showed a very low variant density in the gRNA target sequence. Understanding the genetic variation of gene drive target sequences in natural populations is useful and valuable to guide the implementation of gene drive tools to support current control tools for malaria elimination.

## Materials and methods

### Mosquito sampling

Mosquito sampling was carried out in 8 villages (Bana, Pala, Souroukoudinga, Toma-Ile, Soni, Moara-Grand, Saran and Tosson) in the western part of Burkina Faso (Fig. 5) during 2019. The environmental conditions of these villages are almost similar with an annual rainfall ranging from 700 mm in the north to 1200 mm in the south between May and October. The average annual temperature sits at approximately 27 °C. The sampling area is dominated by agricultural practices especially cereals and cotton cultivation creating suitable environmental niches for the proliferation of mosquito populations. Mosquito samples were collected using pyrethrum spraying catches (PSC) in the rainy season of 2019. Indoor resting mosquitoes were collected early in the morning in 20 randomly selected houses in each village. After collection, mosquitoes were morphologically identified using morphological keys [42, 43] and stored in 80° alcohol for further analyses.

## Molecular analyses

### Mosquito species genotyping

The *An. gambiae* s.l. samples were individually genotyped using the SINE200 protocol described by Santolamazza and collaborators [44] and grouped per species/location. DNA was extracted from individual mosquitoes using a non-destructive extraction method and used to perform a polymerase chain reaction (PCR) for species identification. The polymerase chain reaction (PCR) was performed using the S200 × 6.1F: 5’-TCG-CCT-TAG-ACC-TTG-CGT-TA-3’ and the S200 × 6.1R: 5’-CGC-TTC-AAG-AAT-TCG-AGA-TAC-3’ primers to amplify genomic regions of 479 bp, 249 bp and 223 bp for An. coluzzii, An. gambiae s.s. and An. arabiensis, respectively [44].

### PCR targeting dsx intron 4 – exon 5 boundary and Amplicon sequencing

Genomic DNA was extracted from the pooled mosquitoes using the DNAzol protocol and stored at −20 °C. The extracted DNA was then used to perform PCR for the amplification of the intron 4 – exon 5 boundary (2R: 48714420–48714720) of the *dsx* gene. PCR was performed using KAPA HiFi Hotstart Ready Mix according to the manufacturer’s instructions and the following primers (illumina adapters underlined) previously designed by Hammond and collaborators [21]:

- Forward: 5’-*ACACTCTTTCCCTACACGACGCTCTTCCGATCT*ACTTATCGGCATCAGTTGCG-3’
- Reverse: 5’-*GACTGGAGTTCAGACGTGTGCTCTTCCGATCTGT*GAATTCCGTCAGCCAGCA-3’

A small amount of the PCR product was migrated in an electrophoresis gel and visualized under UV to check the quality of the PCR. The remaining PCR products were purified using the Promega Wizard^®^ SV Gel & PCR clean-up system according to the manufacturer’s instructions. The purity and the amount of DNA in each sample were checked in a Nanodrop. The purity threshold was set between 1.8 and 2 (OD260/280) and the DNA samples whose purity was outside this threshold were removed. The purified DNA samples were adjusted to a concentration of 20 ng/ml in a 25 µl of a nuclease-free water and then shipped for sequencing at Genewiz Amplicon-EZ sequencing.

### Data analyses

#### Amplicon sequencing data

Genomic data were received in FASTQ format from the sequencing company. Various tools including fastp, bwa, samtools, IGV and lofreq were used to handle the data for quality checking, trimming, mapping to the reference genome and variant calling. Quality checking and trimming were performed using fastp v0.23.2 [45]. During this step, the DNA sequences whose length is less than 50 bp and Phred score less than 30 were removed from the data. After quality checking and trimming, the genomic data were mapped to the *An. gambiae* reference genome (AgamP4) [46] using bwa 0.7.17-r1188 [47] and samtools v1.16.6 [48] and stored in bam format. The bam files were indexed and visualized using IGV v17.0.3-internal [49] to check the quality of the mapping to the reference genome. The genetic variants were called from these bam files using the lofreq v2.1.6 program (Wilm et al., 2012) and stored in VCF format [50]. The VCF files were handled in the Jupyter Notebook [52] to analyze the allele frequencies of the identified variants.

#### The Anopheles gambiae 1000 Genomes Consortium data

We also analyzed the spatiotemporal dynamics of the gene drive target sequence variants using the genomic data of the Ag1000G Consortium project phase 3.0, 3.4 and 3.8. These data were from wild caught *An. gambiae* (*An. coluzzii*,* An. gambiae* s.s. and *An. arabiensis*) mosquitoes in 19 African countries (Fig. 1). The dataset of Burkina Faso was from time series sampling of wild *An. gambiae* mosquitoes spanning from 2012 to 2019 collected in three villages (Bana, Pala and Souroukoudinga) (Fig. 5). Full details about the mosquito sampling, sequencing technology, the raw data clean-up and quality control, variant calling, storage of the data and the rights to access these data, have been described on the homepage of MalariaGEN [52]. Python packages such as Malariagen-data [52] and scikit-allel [52] were used to analyze the spatiotemporal distribution of the variants in the gene drive target sequence. We also analyzed the time series variation of the nucleotide diversity (i.e. the average nucleotide differences per site between two randomly selected individuals in the same population) in the gene drive target sequence within the *An. gambiae* populations from 2012 to 2017.

## Supplementary Information

Supplementary Material 1.

Supplementary Material 2.

Supplementary Material 3.

## Data Availability

The raw amplicon sequencing data generated and analyzed during the current study are available from the corresponding author upon reasonable request. Jupyter Notebooks and scripts to reproduce all the analyses, tables and figures are available in the GitHub repository: https://github.com/mkient/dsx_works.git. The SNP and haplotype data from the Ag1000G project are available on the MalariaGEN website and can be accessed using the malariagen_data package. The raw sequences in FASTQ format and the aligned sequences in BAM format were stored in the European Nucleotide Archive (ENA, Study Accession n° PRJEB42254).
