# Correction: Genomic analyses revealed low genetic variation In the Intron-exon boundary of the *doublesex* gene within the natural populations of *An. gambiae* s.l. in Burkina Faso

**DOI:** 10.1186/s12864-025-11515-y

**Published:** 2025-03-31

**Authors:** Mahamadi Kientega, Ioanna Morianou, Nouhoun Traoré, Nace Kranjc, Honorine Kaboré, Odette N Zongo, Abdoul-Azize Millogo, Patric Stephane Epopa, Franck A. Yao, Adrien M G Belem, Austin Burt, Abdoulaye Diabaté

**Affiliations:** 1 Institut de Recherche en Sciences de la Santé, Bobo-Dioulasso, 01 BP 545 Burkina Faso; 2 Department of Life Sciences, Imperial College London, Lon- don, SW7 2AZ UK; 3 Université Nazi BONI, Bobo-Dioulasso, 01 BP 1091 Burkina Faso; 4 University of East Anglia, Norwich Research Park, Norwich, NR4 7UH UK; 5 Institut des Sciences des Sociétés, Ouagadougou, 03 BP 7047 Burkina Faso

**Correction:** ***BMC Genomics*** **25, 1207 (2024)**

10.1186/s12864-024-11127-y

Following publication of the original article it was reported that there was an error in Austin Burt’s name.

It was incorrectly given as: Burt Austin

The correct name is: Austin Burt

The original article has been updated.

