# Supplementary figures and images for: Genomic analyses revealed low genetic variation in the intron-exon boundary of the doublesex gene within the natural populations of An. gambiae s.l. in Burkina Faso
Source: BMC Genomics. 2024 Dec 18;25:1207. doi: 10.1186/s12864-024-11127-y (PMC11657786; doi:10.1186/s12864-024-11127-y)

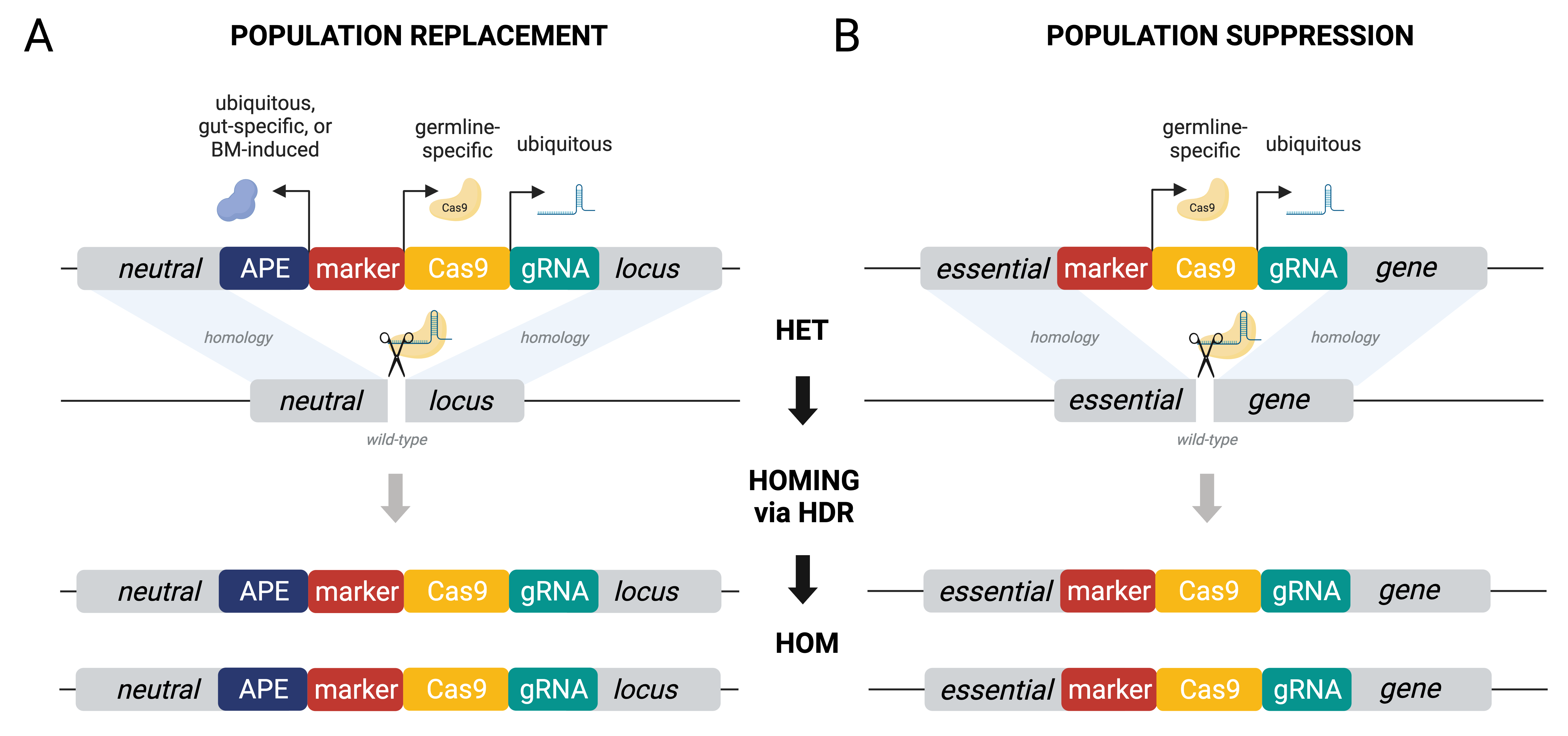

Supplement: Supplementary file 3 — Supplementary Material 3. [file 12864_2024_11127_MOESM3_ESM.png]
